# Supplementary material for: The Systems Biology Research Tool: evolvable open-source software
Source: BMC Syst Biol. 2008 Jun 29;2:55. doi: 10.1186/1752-0509-2-55 (PMC2446383; doi:10.1186/1752-0509-2-55)
Supplement: Additional file 1 — SBRT Archive. An archive of the current version of the Systems Biology Research Tool. [file 1752-0509-2-55-S1.zip › sbrt-1.4.0/doc/developers_guide/api/sbrt/kernel/fba/class-use/IrrevRxnFluxome.html]

Uses of Interface sbrt.kernel.fba.IrrevRxnFluxome


|  |  |  |
| --- | --- | --- |
| **Overview** \| **Package** \| **Class** \| **Use** \| **Tree** \| **Deprecated** \| **Index** \| **Help** | |  |
| PREV   NEXT | **FRAMES**    **NO FRAMES**     **All Classes** |  |


---


## **Uses of Interface sbrt.kernel.fba.IrrevRxnFluxome**

| Packages that use IrrevRxnFluxome | |
| --- | --- |
| **sbrt.kernel.fba** | Provides classes and interfaces for creating objects relevant to Flux Balance Analysis. |
| **sbrt.kernel.fba.comp** | Provides classes and interfaces for performing Flux Balance Analysis computations. |
| **sbrt.kernel.fba.proc** | Provides classes and interfaces for executing Flux Balance Analysis processes. |
| **sbrt.shell.mng.fba** | Provides classes and interfaces for creating Flux Balance Analysis process managers. |
| **sbrt.shell.proc.fba** | Provides classes for converting FBA-related files of different formats. |

Uses of IrrevRxnFluxome in sbrt.kernel.fba

| Classes in sbrt.kernel.fba that implement IrrevRxnFluxome | |
| --- | --- |
| `class` | `IrrevRxnFluxomeV1`             This class is a concrete implementation of IrrevRxnFluxome. |

| Methods in sbrt.kernel.fba that return IrrevRxnFluxome | |
| --- | --- |
| `IrrevRxnFluxome` | `IrrevRxnFluxome.replaceConstraints(FluxConstraints constraints, double constraintTolerance)`             Returns a copy of this fluxome, but with all constraints replaced by those provided. |

| Methods in sbrt.kernel.fba with parameters of type IrrevRxnFluxome | |
| --- | --- |
| `static IrrevRxnFluxomeV1` | `IrrevRxnFluxomeV1.make(java.util.Collection<java.lang.String> reactionNames, IrrevRxnFluxome fluxome)`             Returns an irreversible reaction fluxome composed of the reactions in the provided fluxome with the names in the provided collection. |

Uses of IrrevRxnFluxome in sbrt.kernel.fba.comp

| Methods in sbrt.kernel.fba.comp that return IrrevRxnFluxome | |
| --- | --- |
| `IrrevRxnFluxome` | `AbstractCvComputer.getFluxome()`             Returns the fluxome for which values will be computed. |
| `IrrevRxnFluxome` | `FbaOptComputer.getFluxome()`             Returns the fluxome used by this optimization computer. |
| `IrrevRxnFluxome` | `MsFluxomeReducer.makeFluxome(IrrevRxnFluxome fluxome)`             Returns the sub-fluxome resulting from linear programming. |
| `IrrevRxnFluxome` | `WwFluxomeReducer.makeFluxome(IrrevRxnFluxome fluxome)`             Returns the sub-fluxome resulting from linear programming. |

| Methods in sbrt.kernel.fba.comp with parameters of type IrrevRxnFluxome | |
| --- | --- |
| `SparseDoubleMatrix<?,java.lang.String>` | `SlpCycleComputer.getCycles(IrrevRxnFluxome fluxome)`             Returns the set of all steady state cycles (Type III extreme pathways) contained in the provided fluxome. |
| `SparseDoubleMatrix<?,java.lang.String>` | `CycleComputer.getCycles(IrrevRxnFluxome fluxome)`             Returns the set of all steady state cycles contained in the provided fluxome. |
| `SparseDoubleMatrix<?,java.lang.String>` | `SlpExCurrComputerV2.getMatrix(IrrevRxnFluxome fluxome)`             Returns the extreme current matrix for the provided fluxome. |
| `SparseDoubleMatrix<?,java.lang.String>` | `ExtremeCurrentComputer.getMatrix(IrrevRxnFluxome fluxome)`             Returns the extreme current matrix for the provided fluxome. |
| `SparseDoubleMatrix<?,java.lang.String>` | `SlpExCurrComputerV2.getMatrix(IrrevRxnFluxome fluxome, MutableProgressMonitor pm)`             Returns the extreme current matrix for the provided fluxome. |
| `SparseDoubleMatrix<?,java.lang.String>` | `ExtremeCurrentComputer.getMatrix(IrrevRxnFluxome fluxome, MutableProgressMonitor pm)`             Returns the extreme current matrix for the provided fluxome. |
| `int` | `SlpExCurrComputerV2.getTotal(IrrevRxnFluxome fluxome)`             Returns the total number of "operations" required to compute the extreme currents of the provided fluxome. |
| `int` | `ExtremeCurrentComputer.getTotal(IrrevRxnFluxome fluxome)`             Returns the total number of operations required to compute the extreme currents of the provided fluxome. |
| `IrrevRxnFluxome` | `MsFluxomeReducer.makeFluxome(IrrevRxnFluxome fluxome)`             Returns the sub-fluxome resulting from linear programming. |
| `IrrevRxnFluxome` | `WwFluxomeReducer.makeFluxome(IrrevRxnFluxome fluxome)`             Returns the sub-fluxome resulting from linear programming. |

Constructors in sbrt.kernel.fba.comp with parameters of type IrrevRxnFluxome

`AbstractCvComputer(IrrevRxnFluxome fluxome)`
  
          Constructs a new constraint-variation computer.

`CdHarFluxVectorComputer(IrrevRxnFluxome fluxome,
LinearSystemSolution<java.lang.String> systemSolution)`
  
          Constructs a new random flux vector computer for the provided fluxome.

`FbaOptComputer(IrrevRxnFluxome fluxome,
ProgramSolver<java.lang.Object,java.lang.Object> solver)`
  
          Constructs a new FBA optimization computer from the
provided fluxome and program solver.

`FbaOptComputer(IrrevRxnFluxome fluxome,
ProgramSolver<java.lang.Object,java.lang.Object> solver,
double constraintTolerance)`
  
          Constructs a new FBA optimization computer from the
provided fluxome, program solver, and constraint tolerance.

Uses of IrrevRxnFluxome in sbrt.kernel.fba.proc

| Methods in sbrt.kernel.fba.proc that return IrrevRxnFluxome | |
| --- | --- |
| `IrrevRxnFluxome` | `CatalystDeletionProcess.getFluxome()`             Returns the fluxome used by the reaction deletion computer. |
| `IrrevRxnFluxome` | `CycleIdProcess.getFluxome()`             Returns the fluxome used in this process. |
| `IrrevRxnFluxome` | `ExtremeCurrentProcess.getFluxome()`             Returns the fluxome used in this experiment. |
| `IrrevRxnFluxome` | `RandomEnvGeneratorV1.getFluxome()`             Returns the fluxome for which random objective functions are to be generated. |
| `IrrevRxnFluxome` | `RandomConstraintsGeneratorV1.getFluxome()`             Returns the fluxome for which random objective functions are to be generated. |
| `IrrevRxnFluxome` | `CvCatalystDeletionProcess.getFluxome()`             Returns the fluxome used by the reaction deletion computer. |

| Methods in sbrt.kernel.fba.proc with parameters of type IrrevRxnFluxome | |
| --- | --- |
| `static java.util.Set<java.lang.String>` | `CatalystDeletionProcess.getRxnNames(java.util.Set<java.lang.String> catalysts, IrrevRxnFluxome fluxome)`             Returns the set of names of reactions whose activity depends on the provided set of catalysts. |

Constructors in sbrt.kernel.fba.proc with parameters of type IrrevRxnFluxome

`CycleIdProcess(IrrevRxnFluxome fluxome,
CycleComputer computer)`
  
          Constructs a new steady state cycle identification process.

`ExtremeCurrentProcess(IrrevRxnFluxome fluxome,
ExtremeCurrentComputer computer)`
  
          Constructs a new extreme current experiment.

`RandomConstraintsGeneratorV1(IrrevRxnFluxome fluxome)`
  
          Constructs a new random constraint generator.

`RandomEnvGeneratorV1(IrrevRxnFluxome fluxome)`
  
          Constructs a new random environment generator.

Uses of IrrevRxnFluxome in sbrt.shell.mng.fba

| Methods in sbrt.shell.mng.fba that return IrrevRxnFluxome | |
| --- | --- |
| `static IrrevRxnFluxome` | `FbaOptManager.getFluxome(ManagerInput<java.lang.String,java.lang.String> input, java.io.PrintWriter out)`             Creates and returns a fluxome based on the provided input. |
| `static IrrevRxnFluxome` | `FbaManager.getFluxome(ManagerInput<java.lang.String,java.lang.String> input, java.io.PrintWriter out)`             Opens the file denoted by `FbaManager.RXN_FILE` in the provided manager input and returns the fluxome it contains. |
| `static IrrevRxnFluxome` | `RandomFluxVectorManager.getFluxome(ManagerInput<java.lang.String,java.lang.String> input, java.io.PrintWriter out)`             Creates and returns a fluxome based on the provided input. |
| `static IrrevRxnFluxome` | `FbaOptManager.getFluxome(ManagerInput<java.lang.String,java.lang.String> input, java.io.PrintWriter out, double tolerance)`             Creates and returns a fluxome based on the provided input. |
| `static IrrevRxnFluxome` | `InitialPointManager.getFluxome(ManagerInput<java.lang.String,java.lang.String> input, java.io.PrintWriter out, double tolerance)`             Creates and returns a fluxome based on the provided input. |
| `static IrrevRxnFluxome` | `FbaManager.getFluxome(java.lang.String fileName)`             Returns the fluxome contained in the file with the provided name. |
| `static IrrevRxnFluxome` | `FbaManager.getFluxome(java.lang.String fileName, java.io.PrintWriter out)`             Returns the fluxome contained in the file with the provided name. |
| `static IrrevRxnFluxome` | `FbaManager.getFluxome(java.lang.String rxnFileName, java.lang.String catalystFileName)`             Returns the fluxome contained in the files with the provided names. |
| `static IrrevRxnFluxome` | `FbaManager.getFluxome(java.lang.String rxnFileName, java.lang.String catalystFileName, java.io.PrintWriter out)`             Returns the fluxome contained in the files with the provided names. |

| Methods in sbrt.shell.mng.fba with parameters of type IrrevRxnFluxome | |
| --- | --- |
| `static CdHarFluxVectorComputer` | `CdHarFluxVectorManager.getComputer(ManagerInput<java.lang.String,java.lang.String> input, java.io.PrintWriter out, IrrevRxnFluxome fluxome, double tolerance)`             Creates and returns a coordinate-direction hit-and-run flux vector computer using the provided input. |
| `static MapTextOutputFile<java.lang.String,Interval>` | `RandomConstraintsManager.getOutputFile(ManagerInput<java.lang.String,java.lang.String> input, java.io.PrintWriter out, IrrevRxnFluxome fluxome)`             Returns an empty output file that is suitable for storing multiple sets of flux constraints. |
| `static MapTextOutputFile<java.lang.String,Interval>` | `RandomEnvManager.getOutputFile(ManagerInput<java.lang.String,java.lang.String> input, java.io.PrintWriter out, IrrevRxnFluxome fluxome)`             Returns an empty output file that is suitable for storing multiple environments. |
| `static TextOutputFile<LinearComb<java.lang.String>>` | `RandomObjectiveManager.getOutputFile(ManagerInput<java.lang.String,java.lang.String> input, java.io.PrintWriter out, IrrevRxnFluxome fluxome)`             Returns an empty output file that is suitable for storing randomly generated objective functions. |
| `static TextOutputFile<LinearComb<java.lang.String>>` | `RandomObjectiveManager.getOutputFile(java.lang.String fileName, FileFormat format, java.io.PrintWriter out, IrrevRxnFluxome fluxome)`             Returns an empty output file that is suitable for storing randomly generated objective functions. |
| `static MapTextOutputFile<java.lang.String,Interval>` | `RandomConstraintsManager.getOutputFile(java.lang.String fileName, FileFormat format, java.util.Set<java.lang.String> headers, IrrevRxnFluxome fluxome)`             Returns an empty output file that is suitable for storing multiple sets of flux constraints. |
| `static MapTextOutputFile<java.lang.String,Interval>` | `RandomConstraintsManager.getOutputFile(java.lang.String fileName, java.util.Set<java.lang.String> headers, IrrevRxnFluxome fluxome)`             Returns an empty output file that is suitable for storing multiple sets of flux constraints. |
| `static void` | `FbaManager.writeFluxome(IrrevRxnFluxome fluxome, java.lang.String fileName)`             Writes the provided fluxome to a file with the provided name. |
| `static void` | `FbaManager.writeFluxome(IrrevRxnFluxome fluxome, java.lang.String fileName, java.io.PrintWriter out)`             Writes the provided fluxome to a file with the provided name. |

Uses of IrrevRxnFluxome in sbrt.shell.proc.fba

| Methods in sbrt.shell.proc.fba with parameters of type IrrevRxnFluxome | |
| --- | --- |
| `void` | `SbmlWriter.write(IrrevRxnFluxome fluxome, java.lang.String sbmlFileName)`             Writes the provided fluxome in SBML format to a file with the provided name. |
| `void` | `MetatoolFileWriter.writeFile(IrrevRxnFluxome fluxome, OutputFile<java.lang.String> file)`             Writes a metatool input file. |

---


|  |  |  |
| --- | --- | --- |
| **Overview** \| **Package** \| **Class** \| **Use** \| **Tree** \| **Deprecated** \| **Index** \| **Help** | |  |
| PREV   NEXT | **FRAMES**    **NO FRAMES**     **All Classes** |  |


---
